# Aspiration and Iodine Injection in Catarrh of the Lachrymal Sac

**Published:** 1872-12

**Authors:** 


### ASPIRATION AND IODINE INJECTION IN CATARRH OF THE LACHRYMAL SAC.

M. Verneuil employs the following simple and ingenious method of treating catarrh of the lachrymal sac. He evacuates the contents of the sac by direct puncture with the needle and syringe of Pravaz, whereupon he injects the tincture of iodine. This procedure obviates the danger incurred by the least movement on the part of the patient when iodine was injected directly into the canaliculæ after the old plan. If the contents of the sac cannot be removed by aspiration he forces in a few drops of iodine anyhow, and permits them to remain. This method of treatment is now in practice at the hands of many operators.—*Mouvement Medical, October* 5, 1872.

